# *Chlamydia trachomatis* subverts neutrophil cell death pathways through RIP3 and Mcl-1 manipulation

**DOI:** 10.1128/mbio.02098-25

**Published:** 2025-10-06

**Authors:** Rebecca Koch, Naveen Challagundla, Kathrin Stelzner, Thomas Rudel

**Affiliations:** 1 Chair of Microbiology, Biocenter, University of Wuerzburg, Wuerzburg, Germany; University of California, Berkeley, Berkeley, California, USA

**Keywords:** *Chlamydia*, neutrophil, Mcl-1, RIP3, necroptosis

## Abstract

*Chlamydia trachomatis*, an obligate intracellular pathogen, manipulates host cells to evade immune detection, contributing to sexually transmitted diseases with severe complications. Neutrophils, short-lived effector cells, form the first line of innate immune defense against infection. Here, we demonstrate that *Chlamydia* infection extends the lifespan of human neutrophils, creating a cellular niche for its own survival. Lifespan extension involves the neutrophil PI3K/Akt- and the NF-κB signaling pathways. In addition, infection activates the necroptotic effector receptor-interacting protein kinase 3 (RIP3) without inducing cell death. Instead, RIP3 stabilizes the anti-apoptotic protein Mcl-1, enhancing neutrophil survival. This extended survival of neutrophils correlates with an increased number of infectious *Chlamydia* particles. Mcl-1 plays a critical role in neutrophil survival, lifespan extension, and *Chlamydia* survival. Notably, inhibiting RIP3 reduces Mcl-1 levels in neutrophils without affecting their survival. Under these conditions, however, *Chlamydia* load increases, and the dependence on Mcl-1 is bypassed. Our data reveal a new role for necroptosis in neutrophil defense against intracellular *Chlamydia*, highlighting a complex interplay between RIP3 and Mcl-1 that extends neutrophil lifespan and enhances *Chlamydia* survival within these hostile cells.

**IMPORTANCE:**

This study reveals how *Chlamydia trachomatis*, a common sexually transmitted bacterium, manipulates the body’s first immune responders, the neutrophils, to aid its own survival. Normally short-lived, neutrophils live longer when infected by *Chlamydia*, creating a safe environment for the bacteria. This lifespan extension is driven by specific cell survival signals and a protein called RIP3, which surprisingly does not cause cell death here, but helps stabilize another protein, Mcl-1, that keeps neutrophils alive. Blocking RIP3 reduces Mcl-1, but *Chlamydia* still manages to survive, suggesting it can adapt to changes in the host environment. These findings uncover a new layer of complexity in how our immune system interacts with infections and could inform future strategies for treating *Chlamydia* and similar infections.

## INTRODUCTION

*Chlamydia trachomatis* (*C.t.*) is the leading bacterial cause of sexually transmitted infections (STIs), with nearly 130 million new cases reported annually worldwide (1). Acute infections are often asymptomatic but, if untreated, can result in severe complications, such as pelvic inflammatory disease (PID), ectopic pregnancy, and infertility (2). Histopathological analysis of endometrial tissues from patients with chlamydial PID frequently reveals chronic inflammation and significant neutrophil infiltration (3). Our previous studies demonstrated that *C.t*. can survive within neutrophils, suggesting that these immune cells may act as a reservoir for bacterial survival and dissemination (4). This finding, along with the observed neutrophil aggregation in infected tissues, highlights the critical role of neutrophils in the pathogenesis of *Chlamydia* infections.

As obligate intracellular bacteria with limited coding capacity, *Chlamydia* relies on host cell nutrients for replication (5). The bacteria follow a complex developmental cycle during infection. Infectious, non-dividing elementary bodies (EBs) enter the host cell and reside within a membrane-bound vacuole known as an inclusion. Within hours, EBs transition into replicative, non-infectious reticulate bodies (RBs), which undergo several rounds of division before converting back to infectious EBs. These EBs are then released through either extrusion or host cell lysis (6). Stressors such as antibiotic treatment or metabolic restriction can induce a persistent form of *Chlamydia*, sometimes characterized by enlarged aberrant bodies (ABs). Under favorable conditions, ABs can re-enter the developmental cycle, ultimately leaving the host cell as infectious EBs (7).

The aggressive inflammatory response to infection is a key driver of tissue damage, with neutrophils identified as a primary contributor to *Chlamydia*-induced pathology (8). Strong inflammatory infiltrates have been observed in human cases of endometrial and oviduct infections (3), as well as in animal models of *Chlamydia* infection, where they play a significant role in tissue damage (9, 10).

Neutrophils, or polymorphonuclear leukocytes (PMNs), are the most abundant effector cells recruited to sites of infection (11). These short-lived cells undergo spontaneous apoptosis within 24 h (12). The regulation of neutrophil proliferation and apoptosis is crucial for maintaining homeostasis in multicellular organisms and for effective host defense against bacterial and viral infections. However, many pathogens have evolved strategies to evade immune defenses by delaying neutrophil apoptosis, thereby extending their lifespan, facilitating the formation of replicative niche within them, and enhancing infection (13–17).

Emerging evidence indicates that neutrophil apoptosis is not the only form of programmed cell death; regulated necrosis, or necroptosis, has also been identified as a significant mechanism. Unlike apoptosis, necroptosis is characterized by cell swelling, plasma membrane rupture, and the release of intracellular contents, including damage-associated molecular patterns (DAMPs), which amplify inflammation (18). Necroptosis is triggered by various stimuli, such as tumor necrosis factor α (TNFα), interferon γ, lipopolysaccharide (LPS), and both pathogen- and damage-associated molecular patterns (19). This process is mediated by the necrosome, a protein complex composed of receptor-interacting protein kinase 1 (RIP1), RIP3, and mixed lineage kinase domain-like protein (MLKL) (20). RIP3 is critical for the initiation and precise regulation of necroptosis, while MLKL is responsible for the execution of this cell death mode (21). Subsequent studies have demonstrated that necroptosis plays a crucial role in host defense against pathogens by clearing replication niches and eliminating infected cells, thereby exerting bactericidal effects. However, viruses, parasites, and bacteria have evolved strategies to manipulate this lytic cell death mechanism to establish successful infections (20, 22).

This study aims to enhance our understanding of the molecular pathogenesis and immune evasion strategies employed by *Chlamydia* in infected human neutrophils. We provide evidence that *Chlamydia* infection prolongs neutrophil lifespan, correlating with extended pathogen survival. The pro-survival effects on neutrophils were linked to the activation of PI3K/Akt and NF-κB signaling pathways. The PI3K/Akt pathway was shown to inhibit caspase-3 activity, thereby suppressing apoptosis and stabilizing Mcl-1, a key regulator of neutrophil survival. Additionally, neutrophils were found to initiate necroptosis as a defense mechanism against *Chlamydia*. However, the pathogen appeared to counteract this cell death process by stabilizing Mcl-1, further promoting its survival.

## RESULTS

### *Chlamydia trachomatis* infection extends the lifespan of neutrophils

**Fig 1:**
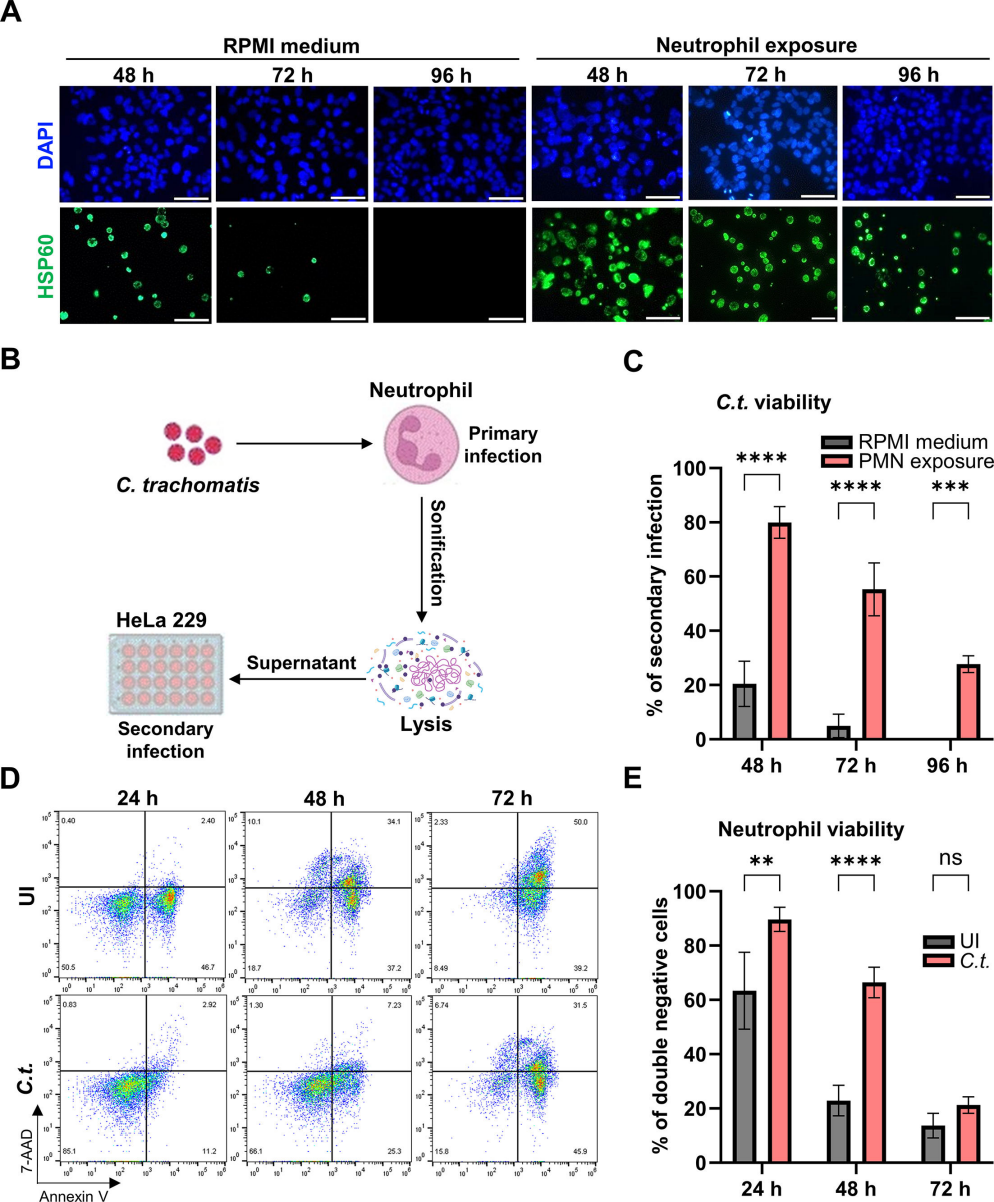
*Chlamydia trachomatis* survives in human neutrophils. (**A**) *C. trachomatis* (*C.t*.) was incubated either in serum-free RPMI 1640 medium or with freshly isolated human neutrophils for the indicated periods. The *C.t.-*containing medium was subsequently transferred to overnight-cultured HeLa cells. The *C.t*.-infected neutrophils were lysed to release intracellular bacteria, and the resulting supernatant was transferred to fresh HeLa cells for secondary infection. At 36 h.p.i. (hours post-infection), cells were fixed with 4% paraformaldehyde and stained for DNA using DAPI (blue) and for *C.t*. using a primary anti-cHSP60 antibody (green). Images were captured using immunofluorescence microscopy (magnification 40×). Scale bar represents 100 µm. (**B**) Schematic representation depicting the flow of infectivity assay. (**C**) The number of cell nuclei and *C.t*. inclusions were counted from six random fields depicted in panel **A**. The graph shows the precentage of secondary infection ± SD, based on samples from three independent donors. (**D**) *C.t*.-infected and uninfected (UI) neutrophils were stained with annexin V and 7-AAD and analyzed for cell survival and death using flow cytometry following incubation for the indicated time periods. Representative dot plots of single cells are shown. (**E**) The graph displays the ratios (%) of viable (annexin V-negative and 7-AAD negative) cells ± SD from three different donors. The significance was assessed by two-way ANOVA with Tukey’s multiple comparisons test (**C**) and (**D**). ns, not significant; **P ≤ 0.01; ***P ≤ 0.001; ****P ≤ 0.0001.

Our previous data show that *Chlamydia trachomatis (C.t*.) downregulate antibacterial activities in human neutrophils, allowing the bacteria to survive for extended periods in these short-lived cells (4). To determine the necessity of host cells for *Chlamydia* to survive and complete its biphasic life cycle, we performed an infectivity assay with and without neutrophils. In this assay, chlamydial particles were exposed to cell culture medium alone or to neutrophils in cell culture medium for different periods and then transferred to HeLa cells to determine infectious EBs (Fig. 1A and B). The results showed that *C.t*. was able to enter neutrophils and survive intracellularly (Fig. S1), remaining infectious for up to 96 h with a secondary infection rate of ~30%. However, *C.t*. could not survive beyond 72 h without a host cell, with only about 5% surviving at that time (Fig. 1C). The survival of *C.t*. in neutrophils declined with the prolongation of the incubation period (Fig. 1A and C). Neutrophils have a relatively short lifespan and undergo spontaneous apoptosis within one or two days under the conditions selected in our experiments. The decline in the infectivity assay may be due to the reduced number of viable neutrophils. However, *C.t*.-infected human blood neutrophils exhibited a significantly extended lifespan and increased resistance to cell death compared to uninfected neutrophils (Fig. 1D and E). To characterize this phenomenon, we performed annexin V/7-AAD assays to distinguish between viable (annexin V-negative/7-AAD-negative), apoptotic (annexin V-positive), and late apoptotic/necrotic (annexin V/7-AAD-positive and annexin V-negative/7-AAD-positive) populations. Uninfected neutrophils initiated apoptosis as early as 24 h, with the majority progressing to late apoptosis/necrosis by 72 h. In contrast, infected neutrophils exhibited delayed apoptosis, starting around 48 h, and retained a significantly higher proportion of viable, double-negative cells (63.3%) compared to the uninfected controls (22.9%) (Fig. 1D and E). This delay of neutrophil cell death may be a key mechanism by which *Chlamydia* prolongs its survival within the host, thereby contributing to the establishment of a successful infection.

### *Chlamydia*-mediated Mcl-1 stabilization is essential for preventing neutrophil death

**Fig 2:**
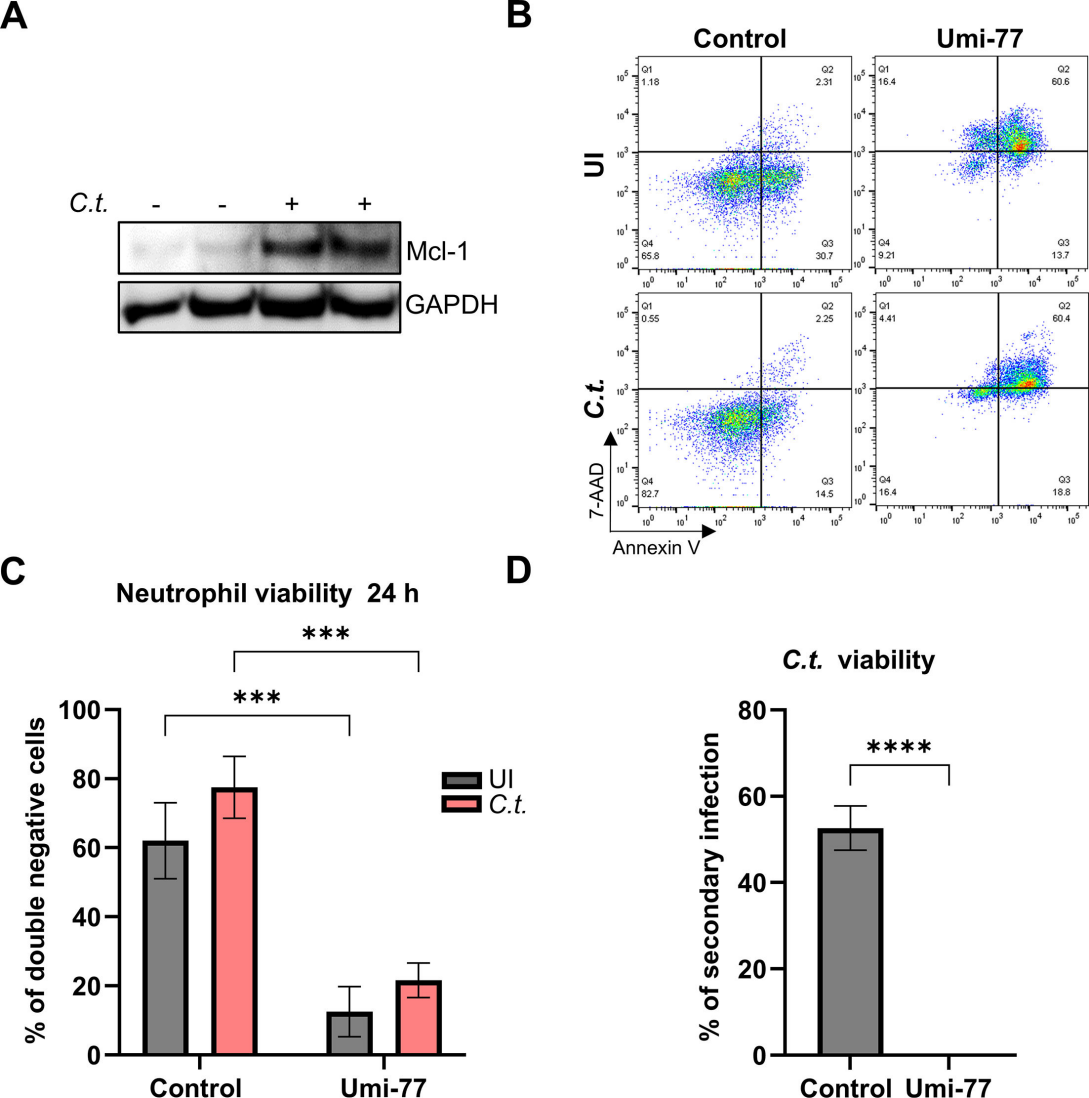
*C.t*.**-**mediated Mcl-1 stabilization is vital for preventing neutrophil death. (**A**) Freshly isolated human neutrophils were infected with *C.t*. (MOI 5) for 48 h. Uninfected cells were used as a control. The cells were lysed and analyzed by SDS-PAGE and Western blot using the antibodies against Mcl-1 (40 kDa) and GAPDH (37 kDa), which served as a loading control. Western blot data represent two technical replicates from a single donor and are representative of results obtained from three independent donors. (**B**) Freshly isolated human neutrophils were treated with the Mcl-1 inhibitor Umi-77 (5 µM) 30 min before *C.t*. infection. Uninfected (solvent-treated) cells were used as a control. At 24 h.p.i., cells were stained with annexin V/7-AAD and analyzed for survival and death by flow cytometry. Representative dot plots are shown. (**C**) The graph displays the ratios (%) of viable (annexin V-negative and 7-AAD negative) cells ± SD from three different donors. (**D**) Untreated and Umi-77-treated (5 µM) infected neutrophils were lysed to release *C.t*. into the supernatant, which was transferred to fresh HeLa cells at 72 h.p.i. The secondary infection was allowed to proceed for 36 h, then the cells were fixed and stained using DAPI and a primary anti-cHSP60 antibody. Images were captured using immunofluorescence microscopy (magnification 40×). The number of nuclei and *C.t*. inclusions from six random fields was quantified and plotted as % of secondary infection. Three different donors were considered. The graph shows mean values of infected cells ± SD. The significance was assessed by two-way ANOVA with Tukey’s multiple comparisons test (**C**) or unpaired *t*-test (**D**). ***P ≤ 0.001; ****P ≤ 0.0001.

The neutrophil life cycle is tightly regulated to maintain host homeostasis, with myeloid cell leukemia-1 (Mcl-1) playing a crucial role in preventing neutrophil apoptosis. Under normal conditions, Mcl-1 is progressively degraded throughout the neutrophil lifespan, leading to spontaneous apoptosis in the absence of external survival signals (23, 24). To understand how *Chlamydia* extends neutrophil lifespan, we examined Mcl-1 expression during infection. Our results revealed an increase in Mcl-1 levels in *C.t*.-infected neutrophils compared to uninfected controls (Fig. 2A). To determine whether Mcl-1 is essential for this effect, we treated neutrophils with Umi-77, a selective Mcl-1 inhibitor, 30 min prior to infection. Mcl-1 inhibition effectively reduced neutrophil survival and reversed *C.t*.-induced neutrophil lifespan extension, leading to a marked increase in cell death (Fig. 2B and C). Additionally, Mcl-1 inhibition strongly reduced *C.t*. survival (Fig. 2D). Neutrophils did not undergo cell death during the 30-minute Umi-77 pre-treatment prior to infection. Even a 2-hour Umi-77 exposure did not compromise neutrophil viability (Fig. S2), indicating that infection could not extend neutrophil lifespan under sustained Mcl-1 inhibition. These findings highlight the critical role of elevated Mcl-1 expression in preventing neutrophil cell death during *Chlamydia* infection.

### Akt and NF-κB signaling mediate enhanced neutrophil and *Chlamydia* survival

**Fig 3:**
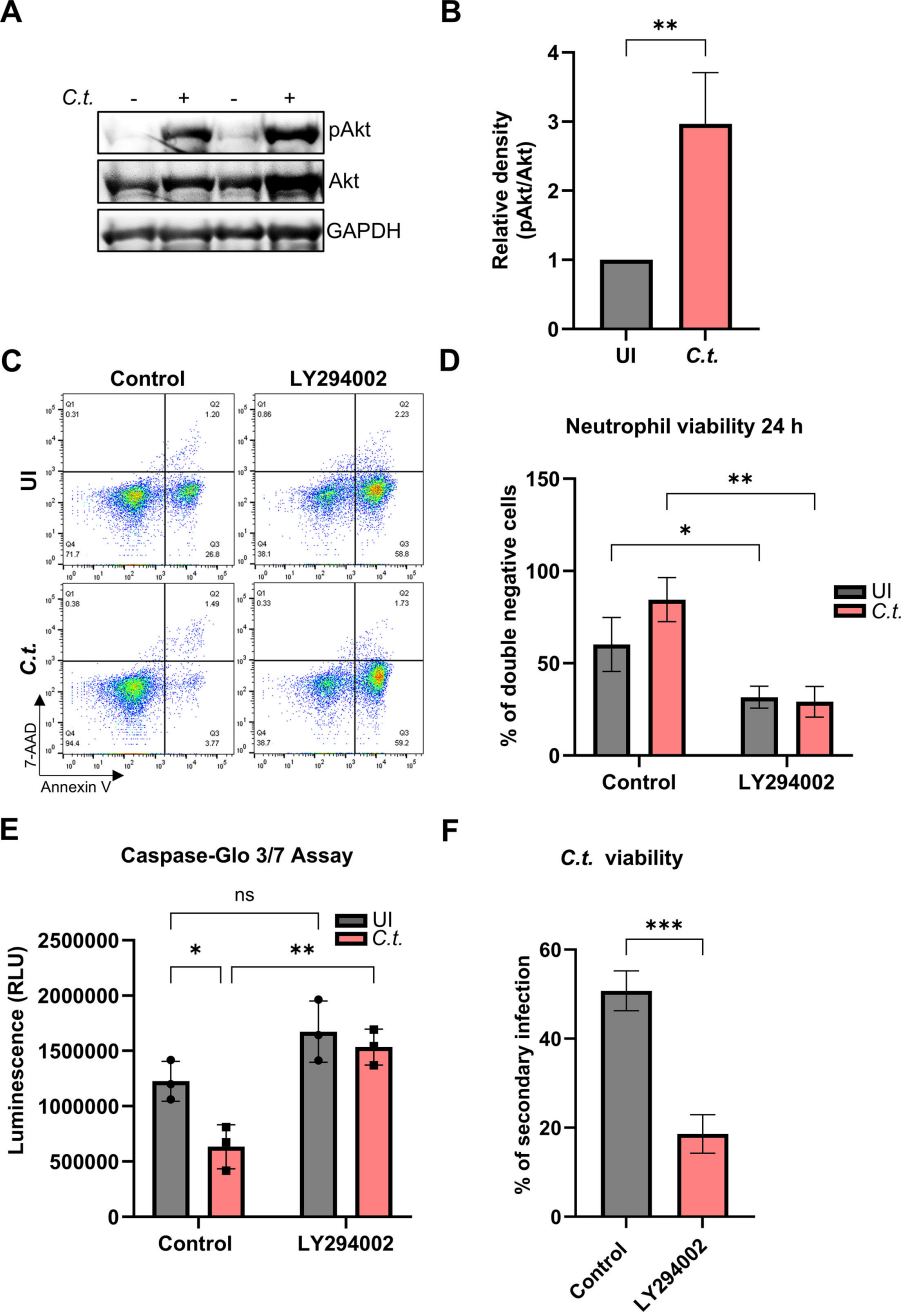
PI3K/Akt signaling mediates enhanced neutrophil and *C.t*. survival. (**A**) Freshly isolated human neutrophils were infected with *C.t*. (MOI 5) for 24 h. Uninfected cells were used as a control. The cells were lysed and analyzed by SDS-PAGE and Western blot using the antibodies against pAkt (60 kDa), Akt (60 kDa), and GAPDH (37 kDa) as loading control. Western blot data shown represent two technical replicates from a single donor and are representative of results obtained from three independent donors. (**B**) Relative blot densities of pAkt/Akt were quantified from three independent experiments (three different donors) using Fiji-Image J (26). The graph shows mean values ± SD. (**C**) Freshly isolated human neutrophils were treated with the PI3K/Akt inhibitor LY294002 (10 µM) 30 min before *C.t*. infection. Uninfected (solvent-treated) cells were used as a control (see Fig. S2). At 24 h.p.i., cells were stained with annexin V/7-AAD and analyzed for survival and death by flow cytometry. Representative dot plots are shown. (**D**) The graph displays the ratios (%) of viable (annexin V-negative and 7-AAD negative) cells ± SD from three different donors. (**E**) Freshly isolated human neutrophils were treated with LY294002 (10 µM) 30 min before *C.t*. infection. Uninfected (solvent-treated) cells were used as control. At 24 h.p.i., caspase-3/7 activity was assessed by monitoring the luminescence (RLU) using the Caspase-Glo 3/7 assay. The graph shows the RLUs ± SD of three independent experiments. (**F**) Untreated and LY294002-treated (10 µM) infected neutrophils were lysed to release *C.t*. into the supernatant, which was transferred to fresh HeLa cells at 72 h.p.i. The secondary infection was allowed to proceed for 36 h when the cells were fixed and stained using DAPI and a primary anti-cHSP60 antibody. Images were captured using immunofluorescence microscopy (magnification 40×). The number of nuclei and *C.t*. inclusions from six random fields was quantified and plotted as % of secondary infection. Three different donors were considered. The graph shows mean values of infected cells ± SD. The significance was assessed by unpaired *t*-test (**B, F**) or two-way ANOVA with Tukey’s multiple comparison test (**D, E**). ns, not significant; *P ≤ 0.05; **P ≤ 0.01; ***P ≤ 0.001.

To elucidate the upstream signals responsible for Mcl-1 stabilization during chlamydial infection, we analyzed the associated signaling pathways. We previously demonstrated that the RAS/MAPK signaling cascade is upstream of Mcl-1 stabilization in *Chlamydia-*infected epithelial cells (25). Our results showed that infection significantly increased Akt phosphorylation in neutrophils, indicating activation of the PI3K/Akt survival pathway in *C.t.-*infected neutrophils (Fig. 3A and B). To further investigate the role of PI3K/Akt in promoting prolonged neutrophil survival, we used PI3K inhibitor LY294002 and showed that inhibiting this pathway effectively eliminated the *Chlamydia*-induced survival advantage in neutrophils, resulting in increased cell death (Fig. 3C). Similarly, neutrophil survival was not affected by the 30-minute pre-treatment with LY294002 prior to infection, as even a 2-hour treatment did not compromise their viability (Fig. S2). Interestingly, unlike the increase in the double positive population observed upon inhibition of Mcl-1 signaling (Fig. 2B), inhibition of the PI3K/Akt pathway increased the apoptotic population (Fig. 3C) and thereby decreased the population of viable neutrophils (Fig. 3D), suggesting that *Chlamydia* infection interfered with apoptotic cell death in neutrophils. This was confirmed by the reduced caspase-3/7 activity in infected compared to uninfected neutrophils, and by the release of the anti-apoptotic block upon PI3K/Akt inhibition (Fig. 3E). Notably, the survival of *Chlamydia* was strongly reduced in neutrophils treated with the PI3K/Akt inhibitor (Fig. 3F), again confirming a connection of neutrophil and *Chlamydia* survival in this assay.

**Fig 4:**
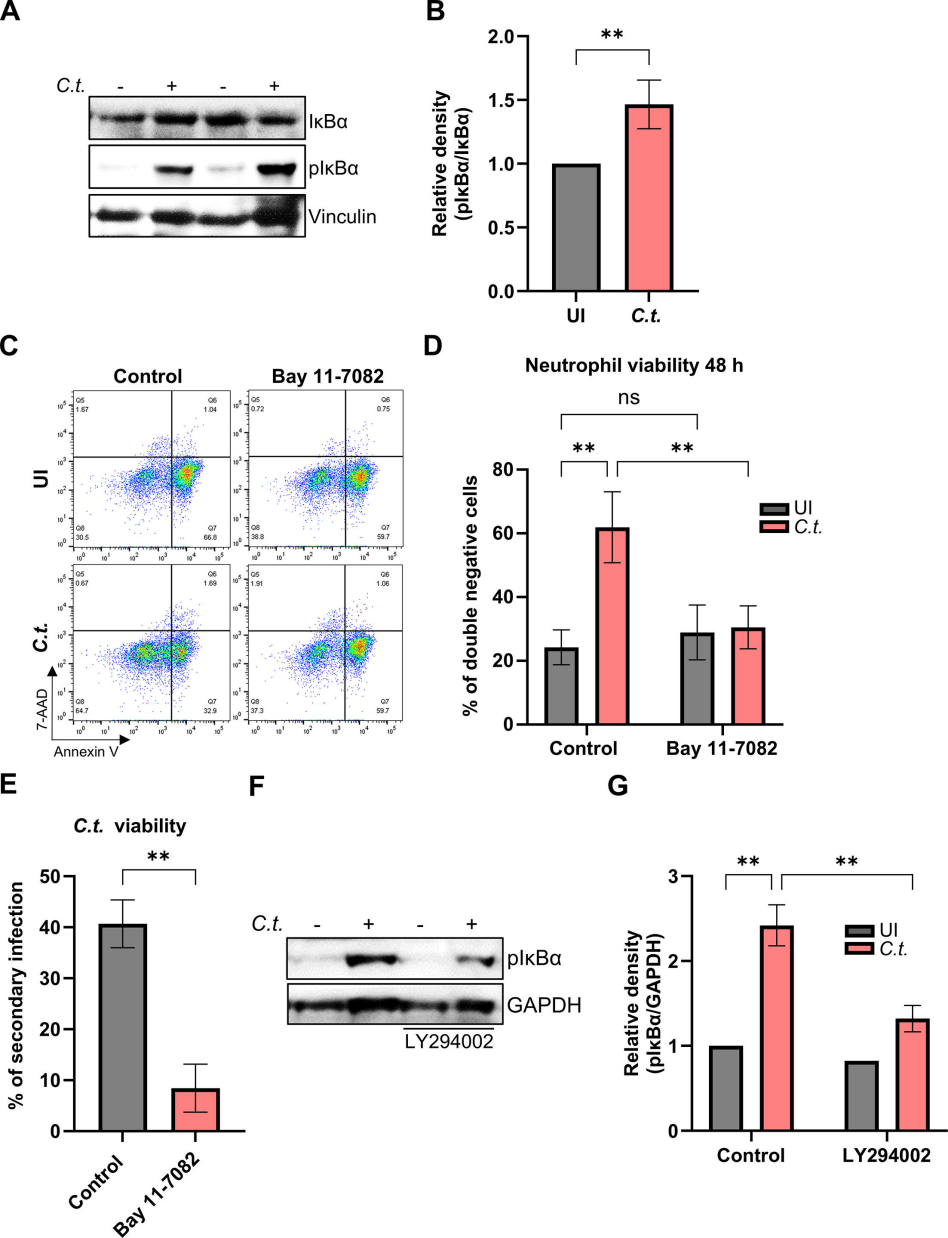
NF-κB signaling mediates enhanced neutrophil and *C.t*. survival. (**A**) Freshly isolated human neutrophils were infected with *C.t*. (MOI 5) for 48 h. Uninfected cells were used as a control. The cells were lysed and analyzed by SDS-PAGE and Western blot using the antibodies against pIκBα (40 kDa), IκBα (40 kDa), and vinculin (116 kDa) as loading control. Western blot data shown represent two technical replicates from a single donor and are representative of results obtained from three independent donors. (**B**) Relative blot densities of pIκBα/IκBα were quantified from three independent experiments (three different donors) using Fiji-Image J. The graph shows mean values ± SD. (**C**) Freshly isolated human neutrophils were treated with the NF-κB inhibitor Bay 11-7082 (0.5 µM) 30 min before *C.t*. infection. Uninfected (solvent-treated) cells were used as a control. At 48 h.p.i., cells were stained with annexin V/7-AAD and analyzed for survival and death by flow cytometry. Representative dot plots are shown. (**D**) The graph displays the ratios (%) of viable (annexin V-negative and 7-AAD negative) cells ± SD from three different donors. (**E**) Untreated and Bay 11-7082-treated (0.5 µM) infected neutrophils were lysed to release *C.t*. into the supernatant, which was transferred to fresh HeLa cells at 72 h.p.i. The secondary infection was allowed to proceed for 36 h when the cells were fixed and stained using DAPI and a primary anti-cHSP60 antibody. Images were captured using immunofluorescence microscopy (magnification 40×). The number of nuclei and *C.t*. inclusions from six random fields was quantified and plotted as % of secondary infection. Three different donors were considered. The graph shows mean values of infected cells ± SD. (**F**) Freshly isolated human neutrophils were treated with LY294002 (10 µM) 30 min before *C.t*. infection. Uninfected (solvent-treated) cells were used as a control. At 48 h.p.i., cells were lysed and analyzed by SDS-PAGE and Western blot using the antibodies against pIκBα (40 kDa) and GAPDH (37 kDa) as loading control. (**G**) Relative blot densities of pIκBα/GAPDH were quantified from two independent experiments using Fiji-Image J. The graph shows mean values ± SD. The significance was assessed by unpaired *t*-test (**B, E**) or two-way ANOVA with Tukey’s multiple comparisons test (**D, G**). ns, not significant; **P ≤ 0.01.

Previous studies have shown that NF-κB regulates cell death in neutrophils (27) and other cells (28). Since activation of PI3K/Akt signaling can be responsible for the activation of NF-κB, and LY294002 has been also shown to interfere with NF-κB signaling (29), we explored the role of NF-κB in *C.t*.-infected neutrophils. Therefore, cells were infected, and the phosphorylation of the inhibitor of NF-κB (IĸBα) was monitored as an indication of NF-κB nuclear translocation and activation. Western blot analysis demonstrated that infection strongly increased phosphorylation of IĸBα (Fig. 4A and B). LY294002 treatment reduced this *C.t.*-induced NF-κB activation (Fig. 4F and G). To test if NF-κB plays a role in the survival of infected neutrophils, we employed BAY 11-7082, an inhibitor of IĸBα-phosphorylation, and the viability of neutrophils was assessed by annexin-V and 7-AAD staining (Fig. 4C). Interestingly, inhibition of the NF-κB pathway increased the apoptotic population of the infected, but not of the uninfected neutrophils (Fig. 4C), thereby decreasing the population of viable infected neutrophils (Fig. 4D). This observation suggests that *Chlamydia* infection specifically induced the activation of NF-κB to interfere with apoptotic cell death in neutrophils. The reduction in the viable infected population had a strong effect on the survival of *Chlamydia* in neutrophils in the presence of the NF-κB inhibitor (Fig. 4E).

### Necroptosis is a critical anti-*Chlamydia* defense pathway in neutrophils

**Fig 5:**
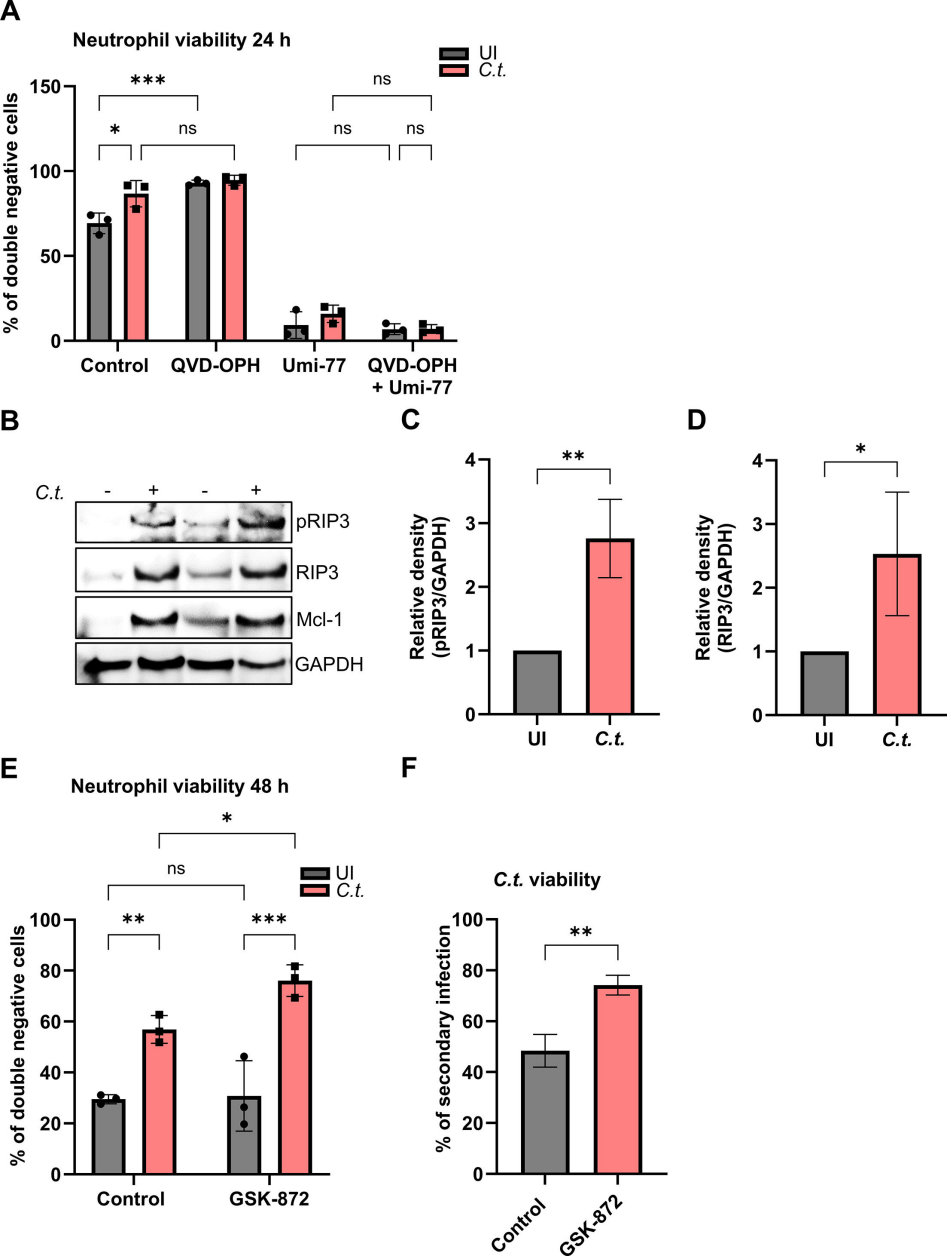
Necroptosis is a central anti-*Chlamydia* defense pathway in neutrophils. (**A**) Freshly isolated human neutrophils were treated with either the pan-caspase inhibitor QVD-OPH (0.5 µM) (30 min before infection) or Umi-77 (5 µM) (at the time of infection) or with both inhibitors. Uninfected (solvent-treated) cells were used as control. At 24 h.p.i., cells were stained with annexin V/7-AAD and analyzed for survival and death by flow cytometry. The graph displays the ratios (%) of viable (annexin V-negative and 7-AAD negative) cells ± SD from three different donors. (**B**) Freshly isolated human neutrophils were infected with *C.t*. (MOI 5) for 48 h. Uninfected cells were used as a control. The cells were lysed and analyzed by SDS-PAGE and Western blot using the antibodies against pRIP3 (46-62 kDa), RIP3 (46-62 kDa), Mcl-1 (40 kDa), and GAPDH (37 kDa). Western blot data shown represent two technical replicates from a single donor and are representative of results obtained from three independent donors. (**C** and **D**) Relative blot densities of (**C**) pRIP3/GAPDH and (**D**) RIP3/GAPDH were quantified from two independent experiments (two different donors) using Fiji-Image J. Both graphs show mean values ± SD. (**E**) Freshly isolated human neutrophils were treated with the RIP3 inhibitor GSK-872 (20 µM) 30 min before *C.t*. infection. Uninfected (solvent-treated) cells were used as a control. At 48 h.p.i., cells were stained with annexin V/7-AAD and analyzed for survival and death by flow cytometry. The graph displays the ratios (%) of viable (annexin V-negative and 7-AAD negative) cells ± SD from three different donors. (**F**) Untreated and GSK-872-treated (20 µM) infected neutrophils were lysed to release *C.t*. into the supernatant, which was transferred to fresh HeLa cells at 72 h.p.i. The secondary infection was allowed to proceed for 36 h when the cells were fixed and stained using DAPI and a primary anti-cHSP60 antibody. Images were captured using immunofluorescence microscopy (magnification 40×). The number of nuclei and *C.t*. inclusions from six random fields was quantified and plotted as % of secondary infection. Three different donors were considered. The graph shows mean values of infected cells ± SD. The significance was assessed by two-way ANOVA with Tukey’s multiple comparisons test (**A, E**) or unpaired *t*-test (**C, D**) and (**F**). ns, not significant; *P ≤ 0.05; **P ≤ 0.01; ***P ≤ 0.001.

We then focused on the cell death pathways orchestrated by Mcl-1, the central regulator of neutrophil survival (Fig. 2B and C). To test if neutrophil cell death induced by Mcl-1 inhibition can be prevented by interfering with apoptosis signaling, we applied the irreversible pan-caspase inhibitor QVD-OPH. Despite caspase inhibition, neutrophil survival was not rescued irrespective of *C.t*. infection (Fig. 5A). QVD-OPH, however, prevented spontaneous apoptosis in uninfected neutrophils (Fig. 5A) as previously described (28). This suggests that mechanisms other than apoptosis contribute to the cell death associated with Mcl-1 inhibition in neutrophils.

We therefore focused on necroptosis, an alternative programmed cell death pathway that has not yet been explored during *Chlamydia* infections. To this purpose, neutrophils were infected for 48 h, and the expression of necroptotic RIP3 and its phosphorylated state was assessed. Western blot analysis of whole-cell lysates displayed that *C.t*. infection increased amounts of both RIP3 and pRIP3 levels, whereas uninfected cells showed little to no phosphorylation (Fig. 5B through D). These observations pointed to necroptosis induction by infection with *Chlamydia* in neutrophils. However, it was not clear whether this cell death mechanism occurred to the benefit of the bacterium or as neutrophil defense strategy to infection. To explore the role of RIP3 signaling in more detail, the viability of infected neutrophils as well as of *Chlamydia* was investigated in the presence of the necroptosis inhibitor GSK-872, which specifically blocks the phosphorylation of RIP3. Annexin V and 7 AAD-staining demonstrated that GSK-872 treatment significantly increased the survival of *C.t.-*infected neutrophils, whereas the inhibitor had no effect on uninfected cells (Fig. 5E). These results indicated necroptosis as a cell death mechanism induced by *Chlamydia* infection in neutrophils. To determine whether the increased neutrophil viability provided a reservoir for bacterial replication, we analyzed the chlamydial burden within these cells. Interestingly, RIP3 inhibition with GSK-872 resulted in an increase in *Chlamydia* numbers (Fig. 5F), suggesting that RIP3 activation occurs as neutrophil defense strategy to eliminate bacteria.

### Elevated Mcl-1 levels sustain neutrophil and *Chlamydia* survival by blocking necroptotic death

**Fig 6:**
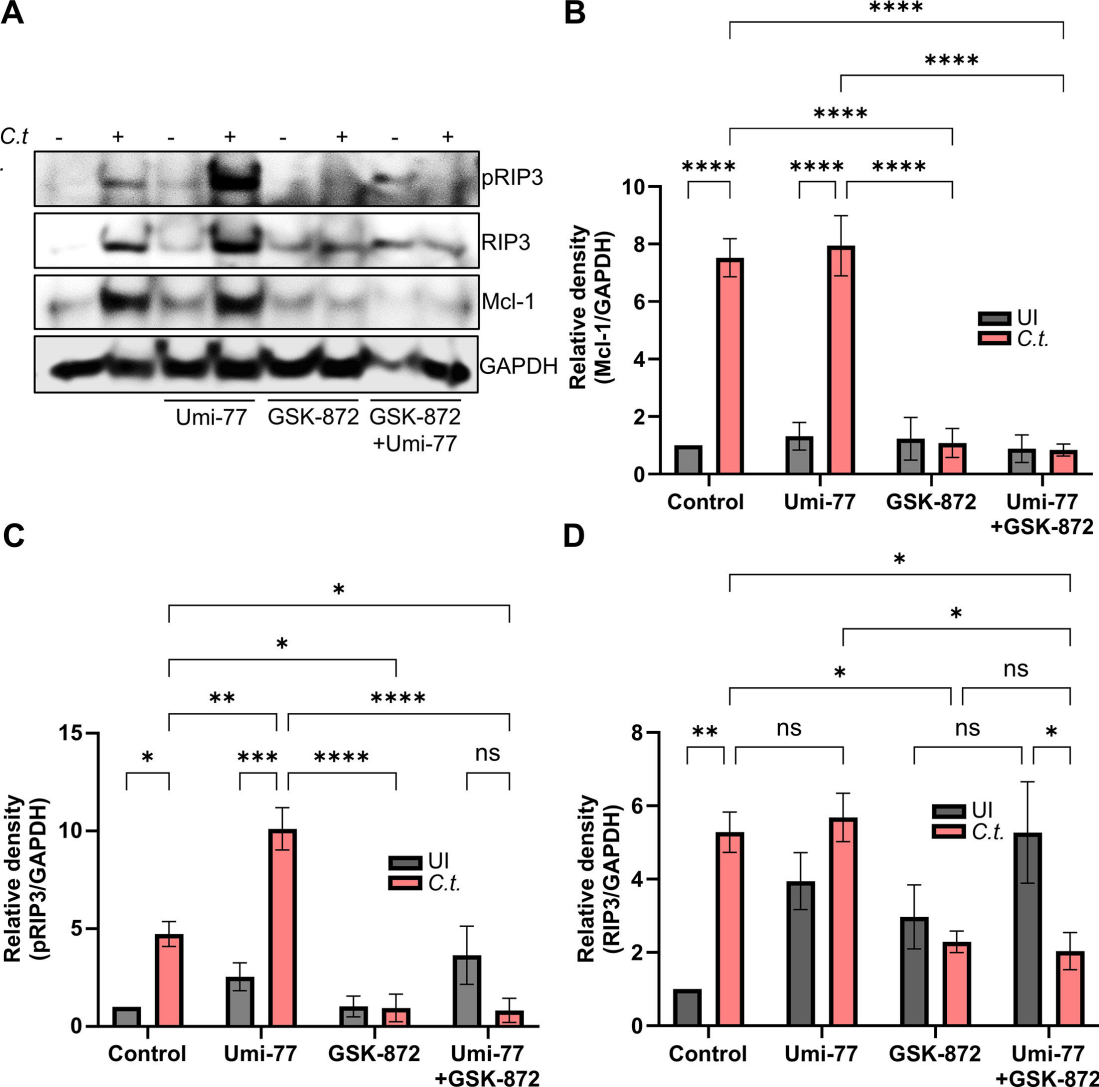
*C.t*.**-**mediated Mcl-1 stabilization correlates with RIP3 activation in neutrophils. (**A**) Freshly isolated human neutrophils were treated with either GSK-872 (20 µM) (30 min before infection) or Umi-77 (5 µM) (at 24 h.p.i.) or with both inhibitors. Uninfected (solvent-treated) cells were used as a control. At 48 h.p.i., cells were lysed and analyzed by SDS-PAGE and Western blot using the antibodies against pRIP3 (46-62 kDa), RIP3 (46-62 kDa), Mcl-1 (40 kDa), and GAPDH (37 kDa) as a loading control. (**B** to **D**) Relative blot densities of (**B**) Mcl-1/GAPDH, (**C**) pRIP3/GAPDH, and (**D**) RIP3/GAPDH were quantified from two independent experiments using Fiji-Image J. All graphs show mean values ± SD. The significance was assessed by two-way ANOVA with Tukey’s multiple comparisons test. ns, not significant; *P ≤ 0.05; **P ≤ 0.01; ***P ≤ 0.001; ****P ≤ 0.0001.

Our data so far have shown that *Chlamydia* infection induces both Mcl-1 and RIP3 expression in neutrophils (Fig. 5B through D and 6A). To further dissect the roles of Mcl-1 and RIP3 in neutrophil survival, we used inhibitors of both proteins separately and in combination. In *C.t.-*infected neutrophils, Mcl-1 inhibition resulted in strongly increased RIP3 phosphorylation, indicating a shift towards necroptosis (Fig. 6A and C). The inhibition of Mcl-1 by Umi-77 did not affect Mcl-1 levels, which were still increased upon infection of neutrophils (Fig. 6A and B). This is in line with the mechanism of Umi-77 which interferes with the interaction of Mcl-1 with the apoptosis effectors Bax and Bak and does not directly affect Mcl-1 levels (30).

**Fig 7:**
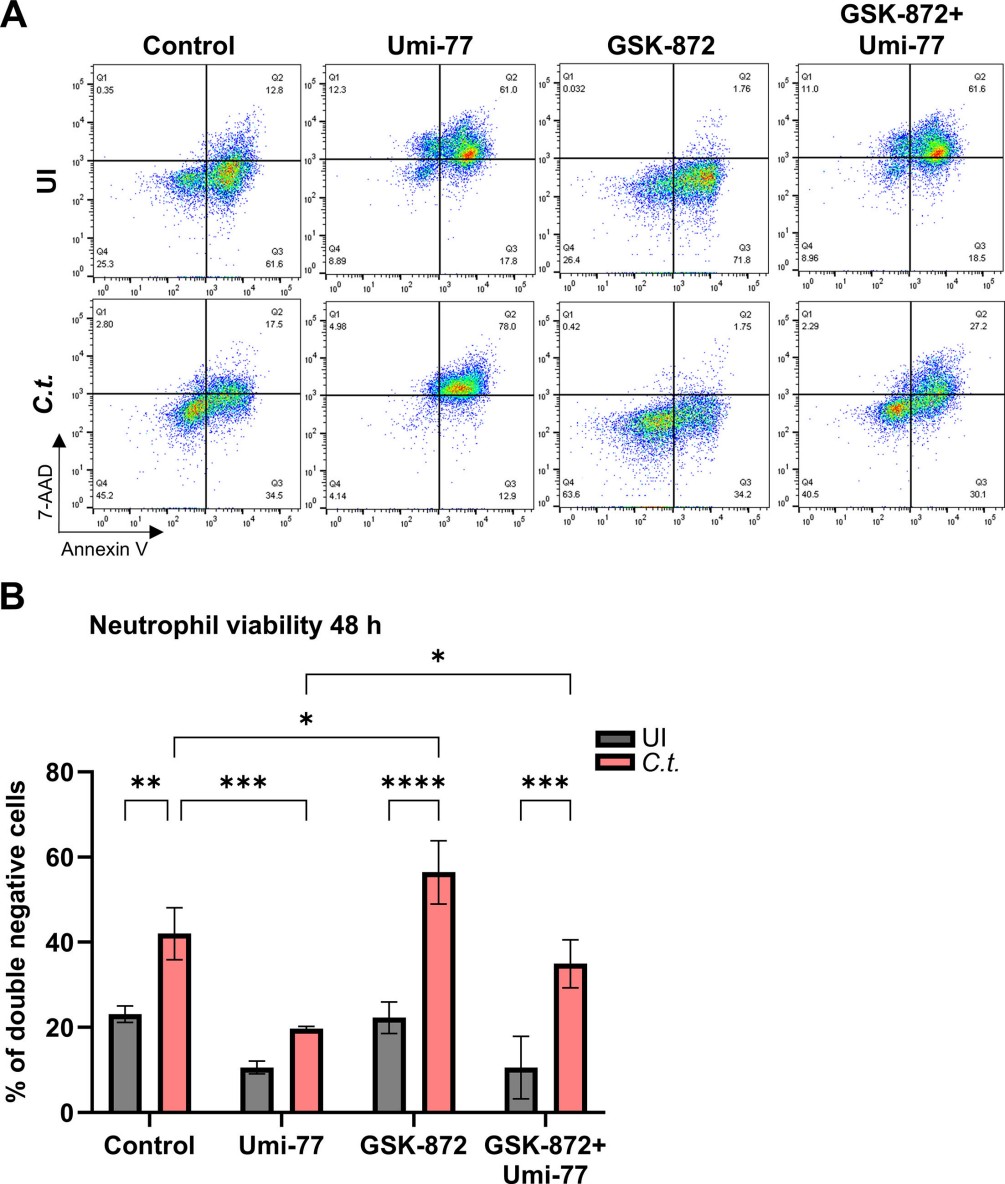
Enhanced Mcl-1 levels maintain neutrophil and *C.t*. survival by blocking RIP3-dependent necroptosis. (**A**) Freshly isolated human neutrophils were treated with either GSK-872 (20 µM) (30 min before infection) or Umi-77 (5 µM) (at 24 h.p.i) or with both inhibitors. Uninfected (vehichle-treated) cells were used as a control (see Fig. S2). At 48 h.p.i., cells were stained with annexin V/7-AAD and analyzed for survival and death by flow cytometry. Representative dot plots are shown. (**B**) The graph displays the ratios (%) of viable (annexin V-negative and 7-AAD negative) cells ± SD from three different donors. The significance was assessed by two-way ANOVA with Tukey’s multiples comparison test. ns, not significant; *P ≤ 0.05; **P ≤ 0.01; ***P ≤ 0.001; ****P ≤ 0.0001.

We then investigated how inhibition of RIP3 phosphorylation affects RIP3 and Mcl-1 levels. GSK-872 reduced Mcl-1 protein levels specifically in *C.t.-*infected, but not in uninfected neutrophils (Fig. 6A and B), indicating that the elevation of Mcl-1 levels upon infection depends on RIP3 activity and the induction of necroptosis. As expected, GSK-872 inhibited the infection-induced phosphorylation of RIP3 (Fig. 6A and C). It also reduced the infection-induced increase in RIP3 levels (Fig. 6A and D), indicating that RIP3 phosphorylation in infected neutrophils also affects RIP3 protein levels. It can be excluded that the lower phosphorylation signals in GSK-872 treated neutrophils depended on lower cell numbers since GSK-872 had no effect on the survival of uninfected neutrophils (Fig. 7A and B), and even a protective effect on *C.t.-*infected neutrophils (Fig. 7A and B).

In the presence of both inhibitors, the strong increase in RIP3 phosphorylation previously observed upon Mcl-1 inhibition alone in infected neutrophils was completely prevented (Fig. 6A and C). Notably, the pronounced induction of neutrophil cell death triggered by the Mcl-1 inhibitor Umi-77 was significantly mitigated by co-inhibition of RIP3 and Mcl-1 (Fig. 7A and B). In contrast, RIP3 inhibition did not protect uninfected neutrophils from Mcl-1 inhibition-induced cell death.

**Fig 8:**
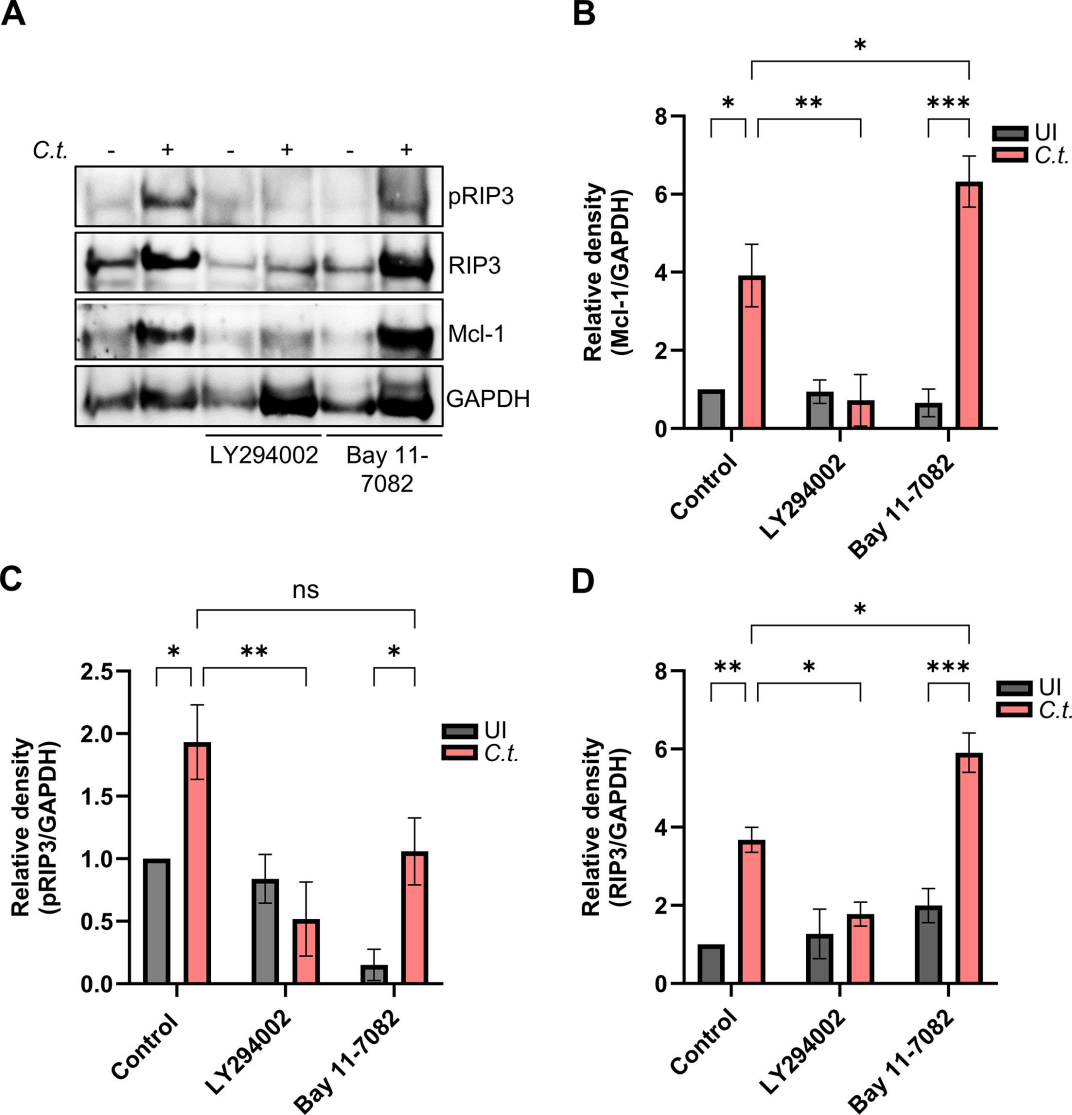
PI3K/Akt and NF-κB signaling affect Mcl-1 and RIP3 levels in *C.t*.-infected neutrophils. (**A**) Freshly isolated human neutrophils were treated with either LY294002 (10 µM) or Bay 11-7082 (5 µM) 30 min before infection. Uninfected (solvent-treated) cells were used as a control. At 48 h.p.i., cells were lysed and analyzed by SDS-PAGE and Western blot using the antibodies against pRIP3 (46-62 kDa), RIP3 (46-62 kDa), Mcl-1 (40 kDa), and GAPDH (37 kDa) as loading control. (**B to D**) Relative blot densities of (**B**) Mcl-1/GAPDH, (**C**) pRIP3/GAPDH, and (**D**) RIP3/GAPDH were quantified from two independent experiments (two different donors) using Fiji-Image J. All graphs show mean values ± SD. The significance was assessed by two-way ANOVA with Tukey’s multiple comparisons test. *P ≤ 0.05; **P ≤ 0.01; ***P ≤ 0.001.

To finally delineate the role of the PI3K/Akt and NF-κB signaling pathways in Mcl-1 stabilization and RIP3 activation, we used the inhibitors LY294002 and Bay 11-7082, respectively, and tested protein levels of pRIP3, RIP3, and Mcl-1 by Western blotting. The inhibition of PI3K/Akt prevented the stabilization of Mcl-1 in infected neutrophils, resulting in a return to baseline levels (Fig. 8A and B), in line with the previous observation of a reduced survival of neutrophils and *Chlamydia* under these conditions (Fig. 3C through E). However, RIP3 activation was also strongly reduced, indicating a role of PI3K/Akt pathways in the induction of necroptosis (Fig. 8A and C). Since inhibition of the PI3K/Akt pathway also led to an increase in caspase-3 activity in both uninfected and infected neutrophils, ultimately resulting in apoptosis (Fig. 3G), the reduced survival of neutrophils and *Chlamydia* most likely depends on a switch in cell death modes between necroptosis and apoptosis under these conditions. These findings emphasize the importance of the PI3K/Akt pathway in preventing both apoptosis through caspase-3 inhibition and necroptosis through Mcl-1 stabilization, thereby promoting neutrophil survival during *Chlamydia* infection.

NF-κB inhibition significantly increased RIP3 level in infected neutrophils (Fig. 8A through C). Conversely, pRIP3 levels showed a downward trend, although not statistically significant (Fig. 8A and D). These findings suggest a correlation between the NF-κB pathway and RIP3 signaling, warranting further investigation to clarify the underlying mechanism.

## DISCUSSION

Working with human neutrophils is challenging due to their short lifespan, donor variability, and the absence of suitable cell or progenitor lines (e.g., the mouse Hoxb8 cell line). These features prevent the use of genetic interference methods and leave inhibitor studies as the only feasible method for functional analysis. In this study, we explored key aspects of *Chlamydia* survival in human neutrophils and its interaction with major cell death pathways. We show that neutrophils support *Chlamydia* survival by extending their lifespan upon infection. Notably, our findings uncover a previously unrecognized role of neutrophil necroptosis in limiting *Chlamydia* survival and reveal infection-driven modulation of necroptosis through Mcl-1 stabilization.

Our data suggest that neutrophils promote infection progression rather than providing effective immune defense against *Chlamydia. Chlamydia* EBs survive significantly longer in the presence of neutrophils than in medium alone, although their survival remains limited by the cells’ lifespan (Fig. 1). This indicates that *Chlamydia* inhibits neutrophil cell death to extend their viability, co-opting neutrophils as a transient niche to sustain bacterial survival.

Notably, this may represent a broader role of neutrophils than previously assumed, as other obligate intracellular bacteria, such as *C. pneumoniae* and *C. psittaci*, also extend their lifespan, and *C. pneumoniae* and *A. phagocytophilum* have even been suggested to replicate within these cells (14, 31, 32).

PI3K/Akt and NF-κB signaling pathways are key regulators of neutrophil survival (27, 33). We confirmed the activation of PI3K/Akt and NF-κB during *C.t*. infection in neutrophils and their essential role in inhibiting neutrophil cell death (Fig. 3A through D and 4A through D), consistent with findings in other *Chlamydia* species (14, 32). Inhibition of these signaling pathways reduced *Chlamydia* survival, possibly due to increased neutrophil cell death. Neutrophils lack several anti-apoptotic Bcl-2 proteins and depend entirely on Mcl-1 expression for survival (34), as shown by severe neutropenia in Mcl-1 conditional knockout (KO) mice (35, 36). Under physiological conditions, Mcl-1 is rapidly degraded via the proteasome (37); however, its stabilization prolongs neutrophil survival (38). Pathogens, such as *Toxoplasma gondii*, *A. phagocytophilum*, and various *Chlamydia* species, exploit similar mechanisms to sustain their host cells (31, 39). Consistently, we observed PI3K-dependent Mcl-1 stabilization in neutrophils during *C. trachomatis* infection (Fig. 2A and 8), correlating with extended neutrophil lifespan.

To investigate the mechanisms behind delayed neutrophil death during *Chlamydia* infection, we examined caspase-3, a key executor of spontaneous neutrophil apoptosis (40), which is known to be inhibited by various pathogens to prolong neutrophil lifespan (14, 31, 41). Similarly, we found that *C.t*. extends neutrophil survival by suppressing caspase-3 activation via PI3K/Akt signaling (Fig. 3F). In contrast, selective inhibition of Mcl-1 markedly reduced neutrophil viability (Fig. 2B and C), underscoring Mcl-1’s essential role in neutrophil survival. Interestingly, while pan-caspase inhibition blocked spontaneous apoptosis in uninfected neutrophils, it failed to rescue viability when Mcl-1 was inhibited, indicating that Mcl-1 inhibition induces a caspase-independent, lytic form of cell death.

Consistent with this, we observed increased levels of RIP3 and phosphorylated RIP3 in infected neutrophils (Fig. 5B through D), and cell death was significantly reduced by the RIP3 inhibitor GSK-872 (Fig. 5E). Importantly, necroptosis inhibition with GSK-872 increased susceptibility to bacterial secondary infection (Fig. 5F), suggesting that necroptosis serves as a previously unrecognized neutrophil defense mechanism during *Chlamydia* infection.

Infection-induced necroptosis as host defense mechanisms has been demonstrated in several other cell types, such as macrophages infected with *Salmonella* (42), epithelial cells infected with *Shigella* (43), and epithelial cells infected with enteropathogenic *E. coli* (EPEC) (44). To our knowledge, this is the first report demonstrating necroptosis as host defense mechanism in infected neutrophils.

**Fig 9:**
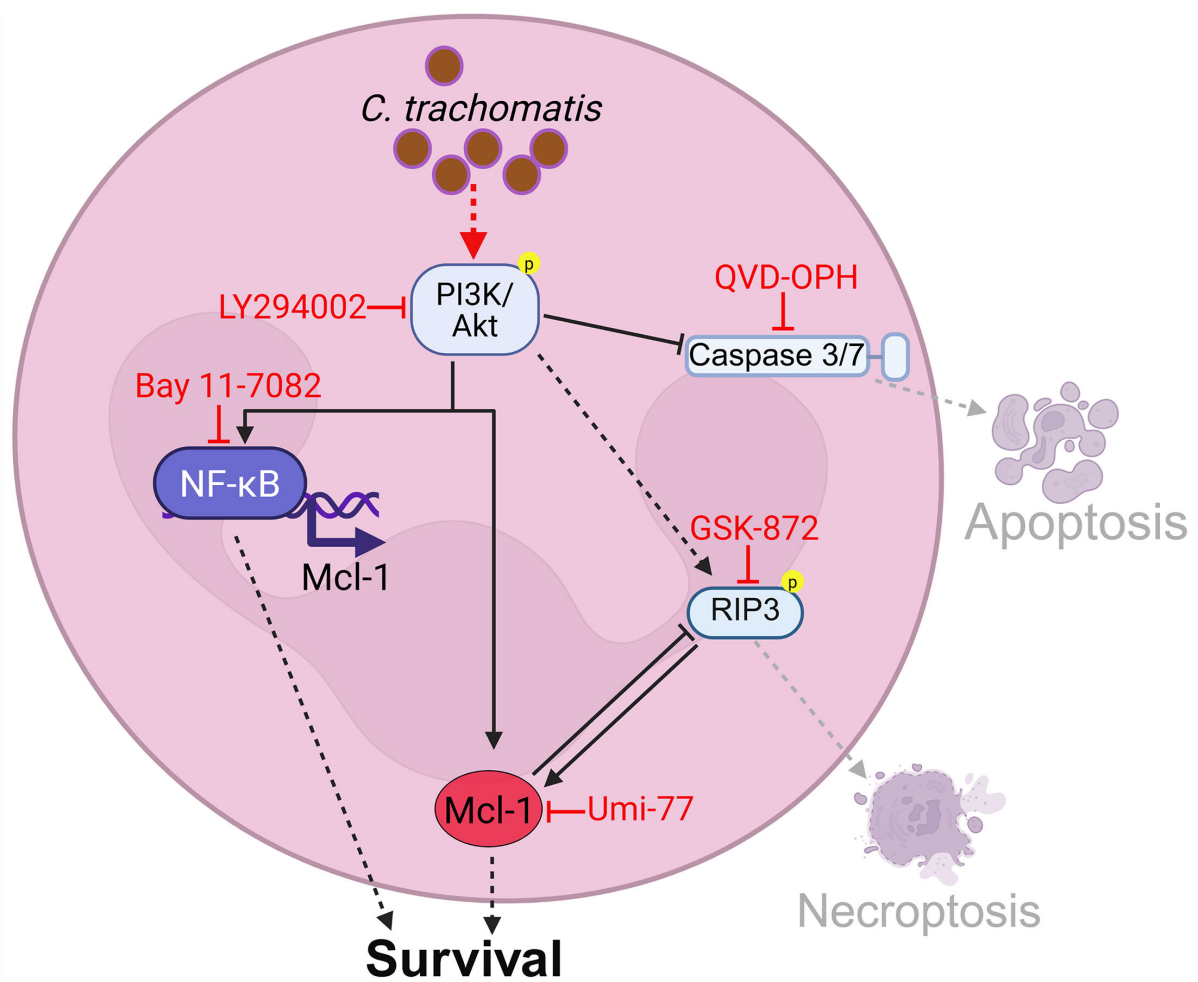
Schematic illustration of *C.t*.-induced survival pathways in neutrophils. *Chlamydia* activates the PI3K/Akt signaling pathway, leading to caspase-3/7 inhibition, NF-κB activation, Mcl-1 stabilization, and RIP3 activation. Caspase-3/7 inhibition suppresses apoptosis, Mcl-1 stabilization counteracts RIP3-mediated necroptosis, collectively promoting neutrophil and *Chlamydia* survival. NF-κB signaling further supports infection by enhancing neutrophil longevity**,** potentially through interference with RIP3 signaling. Although RIP3 is typically associated with necroptosis, its infection-induced expression in neutrophils paradoxically contributes to Mcl-1 stabilization rather than cell death, suggesting a novel pro-survival feedback loop during *C. trachomatis* infection.

Additionally, we observed that inhibition of Mcl-1 with Umi-77 led to visibly increased pRIP3 levels in infected cells (Fig. 6A and C), indicating that Mcl-1 stabilization during infection plays a role in counteracting necroptosis (Fig. 9). Interestingly, inhibiting RIP3 did not result in Mcl-1 stabilization (Fig. 6A and B), suggesting that necroptosis induction contributes to the increased Mcl-1 levels in infected neutrophils. This was further supported by the finding that, unlike in Umi-77-treated infected cells, the phosphorylation of RIP3 was completely reversed and cell death was blocked to a great extent when Mcl-1 inhibition was combined with RIP3 inhibition (Fig. 6C, 7A and B). These data confirm the direct link between RIP3 signaling and Mcl-1 stabilization during infection, highlighting Mcl-1’s protective role in counteracting necroptosis (Fig. 9).

Necroptosis has been initially identified as a form of cell death that occurs upon induction of apoptosis via the TNF receptor and simultaneous inhibition of caspases (45–48). It is tempting to speculate that neutrophils infected by obligate intracellular bacteria are prone to necroptosis induction, considering the potential of these bacteria to interfere with apoptosis—for example, by inhibiting caspases to extend the lifespan of the neutrophil (41, 49, 50). Under this assumption, the intrinsic cell death induction in these cells via the activation of caspases would be counteracted by the intracellular bacteria, which may result in the activation of necroptosis as an alternative pathway to kill the infected neutrophil and protect the host. Given the widespread ability of intracellular pathogens to suppress apoptosis in these cells, necroptosis may have evolved as a crucial host defense strategy to limit pathogen survival. Our findings suggest that *Chlamydia* employs Mcl-1 to suppress necroptosis, effectively counteracting the neutrophil defense strategy. Targeting these signaling pathways could offer new therapeutic opportunities in the future.

## MATERIALS AND METHODS

### Cell culture and bacteria

HeLa cells (ATCC CCL-2.1) were cultured at 37°C with 5% CO₂ in RPMI 1640 medium (Thermo Fisher Scientific) supplemented with 10% fetal bovine serum (FBS; Thermo Fisher Scientific). Chlamydial stocks were prepared as previously described (4).

### Isolation of human neutrophils from peripheral blood

Human neutrophils were isolated using the Ficoll separation method as described previously (51).

### Infection, inhibitors, and secondary infection

Freshly isolated human neutrophils were infected with *C.t*. at an MOI of 5 in 12-well plates containing RPMI 1640 medium and incubated at 37°C with 5% CO₂. Before infection, the wells were coated with 10% plasma in 1× DPBS at 37°C with 5% CO₂ for 1 h to prevent neutrophil activation. After incubation, the wells were washed three times with 1× DPBS. For inhibitor studies, these chemicals and the corresponding amount of DMSO (solvent control) were added to the neutrophils 30 min prior to infection in medium. At specified time points, the cells were lysed for Western blot analysis or further prepared for flow cytometric analysis or the Caspase-Glo 3/7 assay. For secondary infection experiments, inhibitors and the corresponding amount of DMSO (solvent control) were added to the infected neutrophils in culture medium. At defined time points, cells were lysed by sonication, and the lysates were transferred to overnight-grown HeLa cells (1 × 10^5^ per well) in 12-well plates. After a 36-hour incubation to allow inclusion formation (secondary infection), cells were fixed and stained with an anti-HSP60 antibody for immunofluorescence analysis. Images were acquired from six randomly selected fields per well. In each field, the total number of cells (set to 100%) and the number of inclusions (representing infected cells) were counted. The percentage of infected cells was calculated across all fields and plotted as the percentage of secondary infection.

### Antibodies and chemicals

Primary antibodies were purchased from Cell Signaling Technology and include pAkt (Cat#4060), Akt (Cat#9272), Mcl-1 (Cat#5453), pIĸBα (Cat#2859), IĸBα (Cat#4812), pRIP3 (Cat#93654), and RIP3 (Cat#13526). GAPDH was obtained from Santa Cruz (Cat#sc-32233) and vinculin from Sigma-Aldrich (Cat#V9131). The following inhibitors were used in this study: LY294002 (10 µM) (Cell Signaling Technology Cat#9901), Umi-77 (5 µM) (Biozol Cat#HY-18628), Bay 11-7082 (0.5 µM) (Sigma-Aldrich Cat#19542-67-7), QVD-OPH (0.5 µM) (Biozol Cat#HY-12305), and GSK-872 (20 µM) (Cell Signaling Technology Cat#90126).

### Immunofluorescence microscopy

Immunofluorescence staining was performed as described previously (52). After secondary infection of HeLa cells seeded on glass coverslips of 15 mm in diameter, the cells were fixed with 4% paraformaldehyde in 1× DPBS for 45 min. The staining was performed using DAPI (Sigma-Aldrich Cat#D9542) and an anti-cHSP60 (Santa Cruz Cat#sc-57840) primary antibody. Pictures were captured using the LEICA DMR microscope.

### Western blot

Whole-cell lysates were prepared as previously described (53). Briefly, 3 × 10^6^ neutrophils were centrifuged at 500 × *g* for 10 min and resuspended in 700 µL of 10% trichloroacetic acid solution to precipitate the proteins. After keeping the cells for 10 min on ice, neutrophils were centrifuged at 14,000 × *g* at 4°C for 5 min. The pellets were washed two times with 700 µL 100% acetone at 14,000 × *g* at 4°C for 5 min and resuspended in Laemmli buffer. After boiling for 8 min at 95°C, lysates from 1.5 × 10^6^ were used for further procedure which was performed following the protocol as described (54).

### Cell survival assay

To assess neutrophil survival during *C.t*. infection, 10^6^ neutrophils were infected at an MOI of 5 in 12-well plates. At specified time points, annexin V-APC, 7-AAD (BD Pharmingen Cat#550475), and 1 M CaCl_2_ solution were added to the neutrophils in RPMI medium. The cells were incubated in the dark at RT for 10 min and on ice for a further 10 min. Neutrophils were analyzed using an Attune NXT flow cytometer. The percentage of both annexin V- and 7-AAD negative cells was considered as viable cells.

### Caspase 3/7-Glo assay

To determine caspase-3 activity, the Caspase-Glo 3/7 assay (Promega Cat#G8090) was performed according to the manufacturer’s protocol using 5 × 10^4^ neutrophils infected at an MOI of 5 in 96-well microplates (suitable for luminescence assays).

### Statistical analysis

Statistical analysis was performed using the GraphPad Prism 9.3.1 software (GraphPad, San Diego, CA, USA). Data from experiments were analyzed using unpaired Student’s *t*-test between two groups and one-way or two-way ANOVA between multiple groups. Data are shown as mean ± SD. A *P* value of less than 0.05 represented a statistically significant difference.

## Data Availability

The authors declare that all relevant data supporting the findings of this study are available within the paper and its supplemental information.

## SUPPLEMENTAL MATERIAL

Fig. S1 mbio.02098-25-s0001.tif Human neutrophils infected with *Chlamydia trachomatis*.

Fig. S2 mbio.02098-25-s0002.TIF Inhibtor treated human neutrophils analyzed for survival and death by flow cytometry.

ASM does not own the copyrights to Supplemental Material that may be linked to, or accessed through, an article. The authors have granted ASM a non-exclusive, world-wide license to publish the Supplemental Material files. Please contact the corresponding author directly for reuse.
