# Supplementary figures and images for: Chlamydia trachomatis subverts neutrophil cell death pathways through RIP3 and Mcl-1 manipulation
Source: mBio. 2025 Oct 6;16(11):e02098-25. doi: 10.1128/mbio.02098-25 (PMC12607863; doi:10.1128/mbio.02098-25)

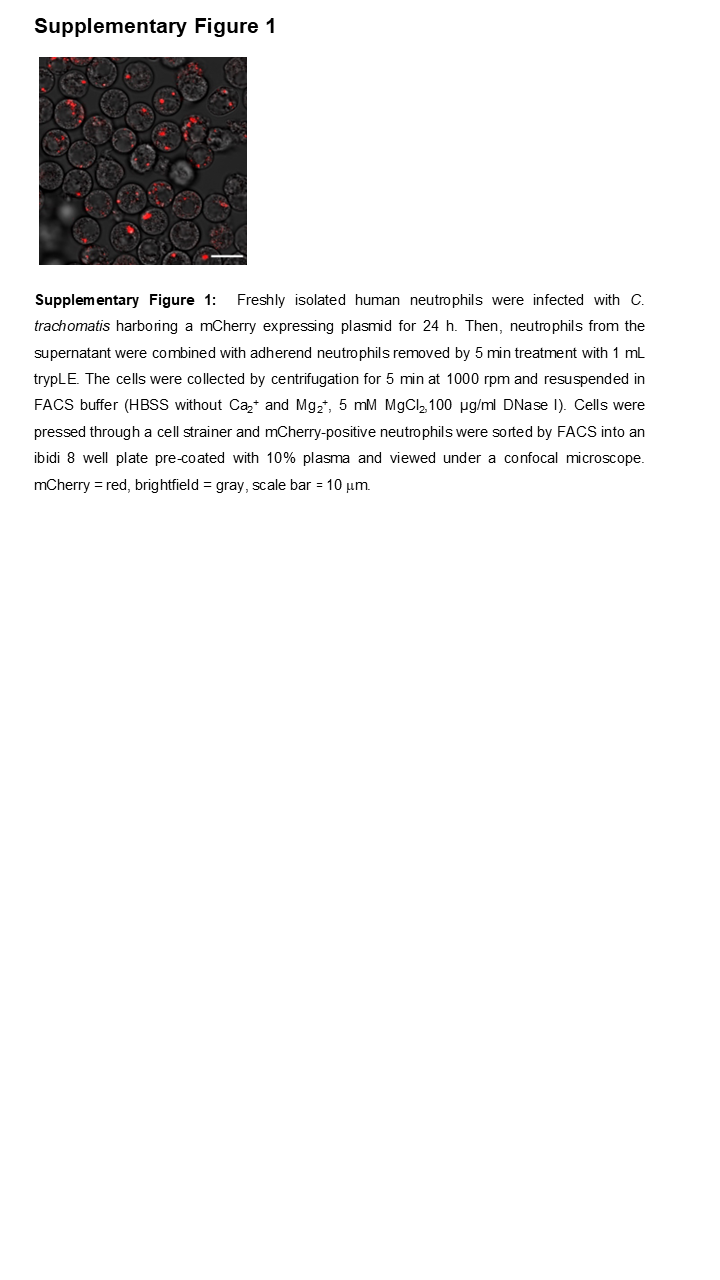

Supplement: Fig. S1 — Human neutrophils infected with Chlamydia trachomatis. [file mbio.02098-25-s0001.tif]

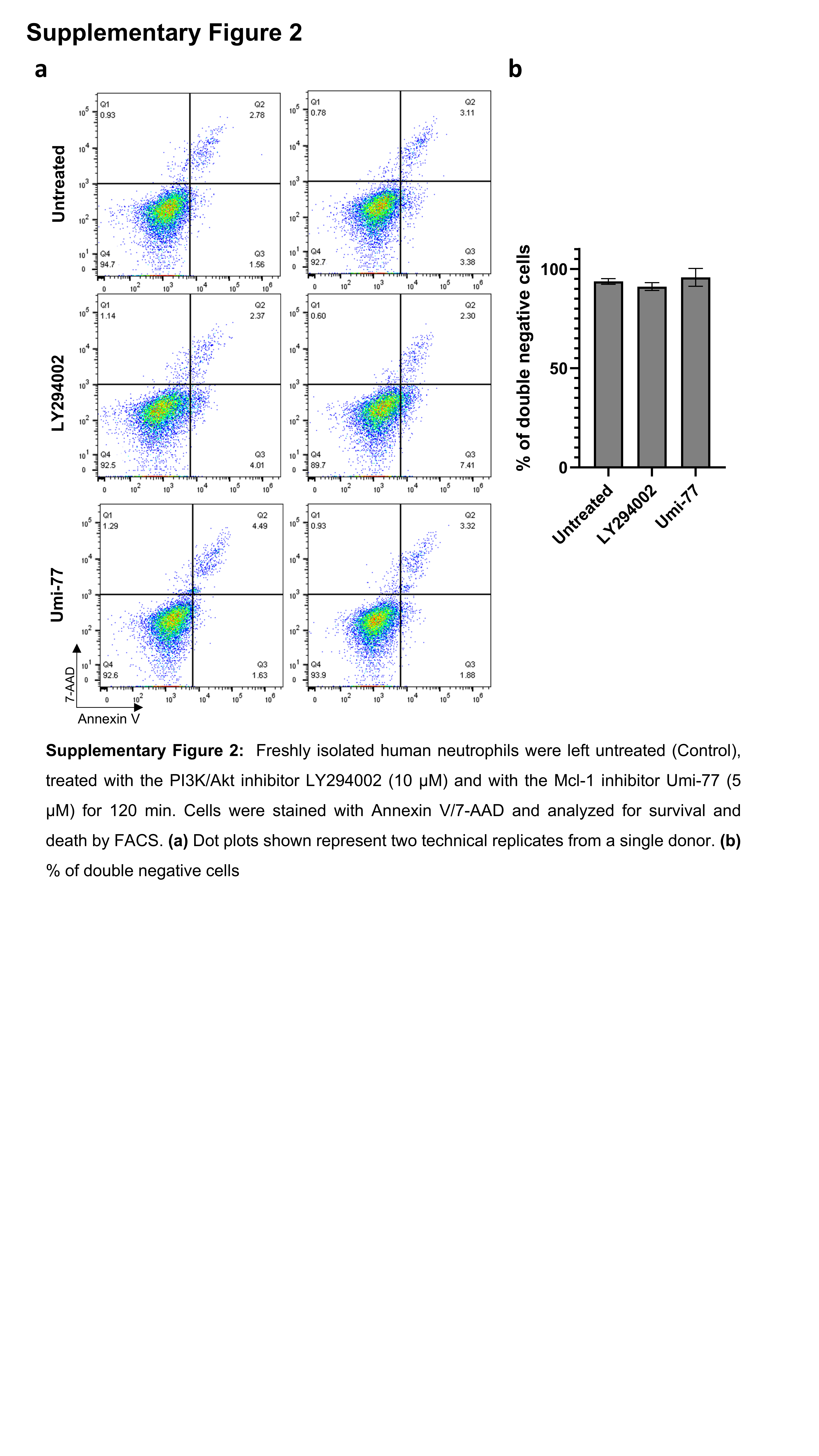

Supplement: Fig. S2 — Inhibtor treated human neutrophils analyzed for survival and death by flow cytometry. [file mbio.02098-25-s0002.tif]
